# Exploiting a chink in the armor: engineering broadly neutralizing monoclonal antibodies for SARS-like viruses

**DOI:** 10.1038/s41392-021-00661-w

**Published:** 2021-06-11

**Authors:** Mirko Cortese, Christopher J. Neufeldt

**Affiliations:** Department of Infectious Diseases, Molecular Virology, Heidelberg University, Heidelberg, Germany

**Keywords:** Microbiology, Structural biology

**Fig. 1:**
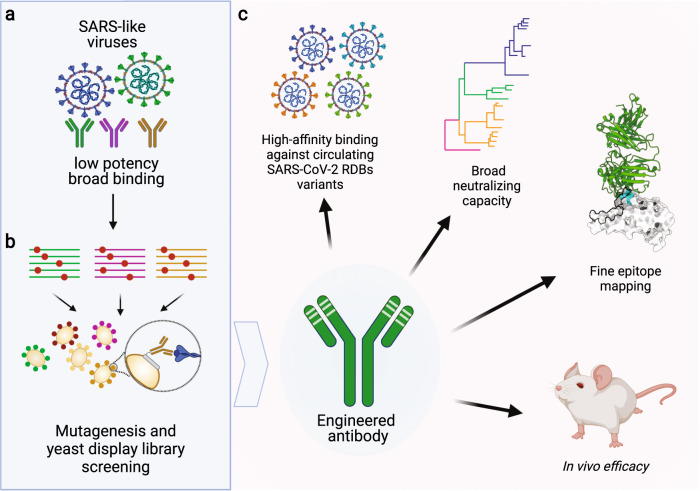
**a** Isolated antibodies from a 2003 SARS survivor showed cross-neutralize activity for SARS-like viruses albeit with low potency. **b** Antibody engineering to increase binding affinity against SARS-CoV-2 S protein using deep mutagenesis and yeast library display. **c** Structural and molecular characterization of engineered antibodies displaying high neutralization breadth and potency against clade-1 sarbecoviruses. Figure created with BioRender.com

In a recent article published in Science, Rappazzo and co-authors^1^ engineered a human monoclonal antibody to bind and neutralize a broad range of SARS-like viruses. The authors used a directed evolution approach with yeast surface display on antibodies isolated from a SARS-CoV-1 survivor, to engineer a broad-spectrum neutralizing antibody with improved neutralization breath, potency, and resistance to viral spike glycoprotein (S protein) mutations (Fig. 1).

In the past 20 years, we have seen three coronavirus outbreaks culminating in the current SARS-CoV-2 pandemic. Vaccination campaigns to control SARS-CoV-2 spread are proceeding at a very different rates across the globe as many countries are facing another rise in SARS-CoV-2 infections. One major contributor to the rising COVID-19 cases is the rapid spread of SARS-CoV-2 variants carrying mutations within the S protein and exhibiting increased infectivity and mortality in the human population.^2^ Although vaccination constitutes the best avenue to curb the SARS-CoV-2 pandemic, vaccine shortages and hesitancy are slowing vaccination programs in many countries. Additionally, people in some risk categories, such as immunocompromised individuals, may elicit suboptimal responses to vaccination and the possibility of vaccine resistant mutants all necessitate the development of complementing therapeutic options to prevent infection or reduce the risk of serious complications.

Highly specific neutralizing monoclonal antibodies (nAbs) targeting the ACE2 receptor binding domain (RDB) of S protein have a good safety profile and a low risk for off-target effects, making for a promising and effective therapeutic treatment for controlling SARS-CoV-2 infection. In addition to preventing SARS-CoV-2 from entering cells through competitive binding to key epitopes required for entry, indirect immunomodulatory Fc-mediated effector functions can contribute to the activation of antiviral cell responses. Although there are still challenges with production and delivery of nAbs, the effectiveness of nAbs in limiting SARS-CoV-2 infection and disease severity has been demonstrated in preclinical settings and shows promise in clinical trials.^3,4^ For clade 1 sarbecoviruses, many of which share a common host receptor, refining nAb epitope specificity and neutralizing capacity also represents one avenue toward development therapeutic agents that have activity against a range of viruses.

In a search for broadly acting countermeasures against coronaviruses, Rappazzo et al. identified three antibodies isolated from a SARS-CoV-1 survivor that exhibited a modest cross-neutralization capacity for a wide range of SARS-like viruses (Fig. 1). Using these initial isolates as a template, the authors employed oligonucleotide-based mutagenesis and yeast homologous recombination to create a library of light and heavy chain mutations that improved binding to the RBD containing subunit of SARS-CoV-2 S protein. They generated several progeny clones with binding profiles from 25-fold to 630-fold higher than the respective parental clones, achieving or surpassing the binding affinities of clinical-stage nAbs. The top three nAb clones (ADG-1, ADG-2, and ADG-3) also exhibited strong neutralizing capacity against SARS-CoV-2 that paralleled benchmark nAbs. Interestingly, there was an observed lack of correlation between affinity and potency, which highlights a strong link between neutralization and epitope specificity rather than binding affinity. When tested against a representative panel of clade 1 sarbecoviruses, ADG-1, ADG-2, and ADG-3 nAb clones all showed strong binding affinity and neutralizing capacity.

To test the therapeutic potential against SARS-CoV-2 variants, the authors tested the nAb clones against 36 single amino acid mutants in the RBD, including the N501Y mutation present in the fast-spreading B.1.1.7 SARS-CoV-2 lineage. While ADG2 and ADG3 variants maintained binding affinity, all but one benchmark clinical SARS-CoV-2 nAb showed a loss of binding for one or more variants.

Cryo-electron microscopy revealed that the epitope recognized by ADG-2 overlapped the hACE2-binding site. A combined fine mutational analysis revealed that mutations in 4 residues of the S protein, which cluster within the cleft formed by the antibody light and heavy chain, can abrogate ADG-2 binding. Notably, 3 of these mutations also disrupt the binding between the S protein and the host hACE2 receptor. These findings explain the conservation of these residues among coronaviruses that rely on ACE2 for entry and suggest a reason that mutations at those positions are largely absent among circulating SARS-CoV-2 variants. Altogether the molecular and structural data provide an explanation for ADG-2 binding breadth by tightly linking epitope binding to essential residues required for hACE2 interaction.

Furthermore, ADG-2 nAbs were effective in triggering Fc-mediated effector functions for NK cell activation and complement deposition, which also surpassed the benchmark SARS-CoV-2 nAbs and both prophylactic and therapeutic administration of ADG-2 reduced disease burden in mice infected with mouse adapted SARS-CoV-1 and SARS-CoV-2 viruses.

The race to develop effective treatments against SARS-CoV-2 infection and COVID-19 is currently challenged by the spread of SARS-CoV-2 variants harboring several mutations in the RDB that render the virus more resistant to monoclonal nAbs that target ancestral variants.^5^ While approved vaccines seems to be equally efficacious in preventing severe disease and death upon infection with the current SARS-CoV-2 variants, there is a risk that new variants might acquire additional mutations leading to vaccine resistance. Moreover, surveillance of zoonotic reservoirs suggests that spill-over of SARS-like viruses will likely occur in the future for which the plethora of newly developed therapeutics will be ineffective. Therefore, there is an urgent need for the development of broadly acting antiviral therapies to combat both SARS-CoV-2 genetic drift as well as emerging SARS-like viruses. The broad-neutralization and in vivo efficacy displayed by ADG-2 clearly demonstrate the validity of the approach by Rappazzo and co-authors.

The major challenges reside now in the clinical application of these therapies. Given that several circulating SARS-CoV-2 variants can escape existing polyclonal antibody responses, it is possible that mutations will arise even in conserved epitopes. One way to increase the efficacy, as well as limit mutational escape, will be to use a combination of nAbs that target multiple viral protein domains. Additionally, it will be important to precisely determine the individuals that will most benefit from nAb therapy as well as establishing the exact timing and period of protection for treatment regimens.

This study not only provides a promising therapeutic candidate against SARS-like viruses, but also identifies an essential epitope within the RBD that could be exploited for the development of future treatments or in structure driven vaccine design. This work represents important steps forward in identifying and targeting conserved receptor binding epitopes, or as the authors themselves indicate, the “Achille´s heel for clade 1 sarbecoviruses”.
